# Functional relationships between recessive inherited genes and genes with de novo variants in autism spectrum disorder

**DOI:** 10.1186/s13229-020-00382-x

**Published:** 2020-10-06

**Authors:** Lin Wang, Yi Zhang, Kuokuo Li, Zheng Wang, Xiaomeng Wang, Bin Li, Guihu Zhao, Zhenghuan Fang, Zhengbao Ling, Tengfei Luo, Lu Xia, Yanping Li, Hui Guo, Zhengmao Hu, Jinchen Li, Zhongsheng Sun, Kun Xia

**Affiliations:** 1 Center for Medical Genetics and Hunan Key Laboratory of Medical Genetics, School of Life Sciences, Central South University, Changsha, Hunan China; 2 Reproductive Medicine Center, Xiangya Hospital, Central South University, Changsha, Hunan China; 3 National Clinical Research Center for Geriatric Disorders, Department of Geriatrics, Xiangya Hospital, Central South University, Changsha, Hunan China; 4 Department of Obstetrics and Gynecology, The First Affiliated Hospital of Anhui Medical University, No 218 Jixi Road, Hefei, 230022 Anhui China; 5 NHC Key Laboratory of Study On Abnormal Gametes and Reproductive Tract (Anhui Medical University), No. 81 Meishan Road, Hefei, 230032 Anhui China; 6 Institute of Genomic Medicine, Wenzhou Medical University, Wenzhou, Zhejiang China; 7 Beijing Institutes of Life Science, Chinese Academy of Sciences, Beijing, China; 8 CAS Center for Excellence in Brain Science and Intelligences Technology (CEBSIT), Shanghai, China; 9 School of Basic Medical Science, Central South University, Changsha, Hunan China

**Keywords:** Autism spectrum disorder, Recessive inherited variant, De novo variant, Expression pattern, Functional network

## Abstract

**Background:**

Both de novo variants and recessive inherited variants were associated with autism spectrum disorder (ASD). This study aimed to use exome data to prioritize recessive inherited genes (RIGs) with biallelically inherited variants in autosomes or X-linked inherited variants in males and investigate the functional relationships between RIGs and genes with de novo variants (DNGs).

**Methods:**

We used a bioinformatics pipeline to analyze whole-exome sequencing data from 1799 ASD quads (containing one proband, one unaffected sibling, and their parents) from the Simons Simplex Collection and prioritize candidate RIGs with rare biallelically inherited variants in autosomes or X-linked inherited variants in males. The relationships between RIGs and DNGs were characterized based on different genetic perspectives, including genetic variants, functional networks, and brain expression patterns.

**Results:**

Among the biallelically or hemizygous constrained genes that were expressed in the brain, ASD probands carried significantly more biallelically inherited protein-truncating variants (PTVs) in autosomes (*p* = 0.038) and X-linked inherited PTVs in males (*p* = 0.026) than those in unaffected siblings. We prioritized eight autosomal, and 13 X-linked candidate RIGs, including 11 genes already associated with neurodevelopmental disorders. In total, we detected biallelically inherited variants or X-linked inherited variants of these 21 candidate RIGs in 26 (1.4%) of 1799 probands. We then integrated previously reported known or candidate genes in ASD, ultimately obtaining 70 RIGs and 87 DNGs for analysis. We found that RIGs were less likely to carry multiple recessive inherited variants than DNGs were to carry multiple de novo variants. Additionally, RIGs and DNGs were significantly co-expressed and interacted with each other, forming a network enriched in known functional ASD clusters, although RIGs were less likely to be enriched in these functional clusters compared with DNGs. Furthermore, although RIGs and DNGs presented comparable expression patterns in the human brain, RIGs were less likely to be associated with prenatal brain regions, the middle cortical layers, and excitatory neurons than DNGs.

**Limitations:**

The RIGs analyzed in this study require functional validation, and the results should be replicated in more patients with ASD.

**Conclusions:**

ASD RIGs were functionally associated with DNGs; however, they exhibited higher heterogeneity than DNGs.

## Background

Autism spectrum disorder (ASD) is an early-onset neurodevelopmental disorder with a global prevalence of 1% and is clinically diagnosed based on social impairment, repetitive behaviors and restricted interests [1]. In addition to these core symptoms, other variable traits in patients with ASD include neuropsychiatric comorbidities such as intellectual disability (ID) and developmental delay [2], leading to marked clinical heterogeneity. ASD is highly heritable, indicating that genetic factors play vital roles in its pathogenesis [3]. Microarray technology [4–8], whole-exome sequencing (WES) [9–19], and whole-genome sequencing [20–28] have been widely used in genetic studies of large ASD cohorts. Numerous de novo variants (DNVs), particularly protein-truncating variants (PTVs), have been detected, and several exome-wide significant genes with DNVs (DNGs) have been elucidated, contributing to a better understanding of the genetic causes of ASD. In our previous studies, we focused on DNVs to explore the genetic architecture and genotype–phenotype correlations in Chinese ASD patients [29–31], demonstrating that analyzing DNG-expression patterns and functional networks could provide clues to elucidate the ASD etiology and subtypes [32–34].

Although DNVs are clearly important in ASD, relatively few exome studies have explored rare inherited variants in ASD. Krumm et al. [35] reported significant maternal transmission bias of inherited truncating variants in male ASD cases. Additionally, studies on both consanguineous and nonconsanguineous families identified several recessive inherited genes (RIGs) with rare biallelically inherited variants in autosomes or X-linked inherited variants in males that are associated with ASD [36–38]. Specifically, Chahrour et al. [36] performed WES for 16 nonconsanguineous families and identified four RIGs (*UBE3B*, *CLTCL1*, *NCKAP5L*, and *ZNF18*). Yu et al. [37] performed WES for multiple consanguineous families and identified six RIGs (*AMT*, *PEX7*, *SYNE1*, *VPS13B*, *PAH*, and *POMGNT1*)*.* Lim et al. [38] identified genes with recessive inherited PTVs using WES data from 933 ASD cases and 869 controls, estimating that complete knockouts of autosomal and X-linked genes accounted for 3% of all ASD cases and 2% of male ASD cases, respectively. Recently, Doan et al. [39] reanalyzed WES data of individuals with ASD from the Autism Sequencing Consortium (ASC), demonstrating that biallelically autosomal PTVs and deleterious missense (Dmis) variants were present in 5% of all ASD cases and 10% of female ASD cases. Moreover, they identified 41 autosomal ASD-associated RIGs [39]; however, recessive X-linked genes were not included in their analysis.

**Fig. 1:**
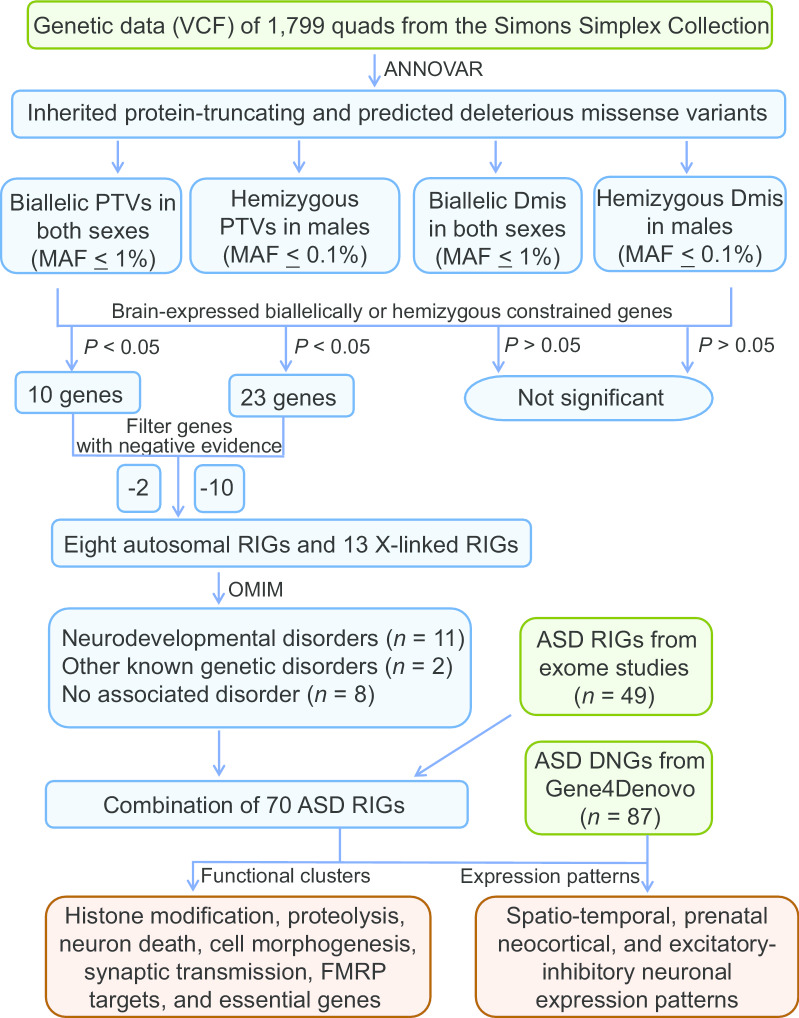
Study workflow. The study included six phases: (1) genetic data collection, (2) identification and annotation of inherited variants, (3) burden analysis, (4) prioritization of ASD-associated RIGs, (5) RIG/DNG functional network analysis, and (6) RIG/DNG brain-expression analysis. ASD, autism spectrum disorder; Dmis, deleterious missense variants; DNGs, genes with de novo variants; PTVs, protein truncating variants; RIGs, recessive inherited genes

To provide resources for the study of ASD genetics, the Simons Simplex Collection (SSC) [40] established a permanent repository of quad samples, each comprising of one ASD proband, one unaffected sibling, and their unaffected parents. To better understand the contributions of RIGs to ASD, we performed genetic burden analysis on biallelically and X-linked inherited coding variants in 1799 quads from the SSC, prioritized a total of 21 candidate RIGs, and investigated the functional relationships between RIGs and DNGs based on functional clusters and expression patterns in the human brain (Fig. 1).

## Materials and methods

### Annotation and identification of inherited variants

Genetic data in VCF format (SSC_WES_3), and the corresponding phenotypic information were downloaded from the SFARI Base (https://www.sfari.org/resource/sfari-base/). Only coding variants with sequencing depth > 20, genotype quality > 50, and call rate > 0.9 were retained for further analysis. Quad samples containing a proband, an unaffected sibling, and their unaffected parents were analyzed. Informed consent was provided in the original study [15]. Comprehensive annotation of all variants was performed by ANNOVAR [41] and VarCards [42], as previously described [29, 32, 33]. Annotation included gene regions, amino acid alterations, mutation effects, GenBank mRNA accession numbers, and minor allele frequencies (MAFs) in the Genome Aggregation Database (gnomAD database, https://gnomad.broadinstitute.org/) [43]. All coding variants were classified into five functional categories: (1) PTVs, including alternative splice variants (defined as variants that occurred within ± 2 bp of an exon–intron boundary), stop-gain and stop-loss single-nucleotide variants, and frameshift indels; (2) Dmis variants with ReVe scores > 0.7 [44]; (3) tolerated missense variants with ReVe scores ≤ 0.7; (4) synonymous variants; and (5) non-frameshift indels.

For the 1,799 quads, we focused on biallelically inherited variants in autosomes with MAF ≤ 1% and X-linked inherited variants in males with MAF ≤ 0.1% based on the SSC cohort and the gnomAD database. For all probands and siblings, we counted the number of samples carrying inherited variants and classified them in the following six groups: (1) biallelically inherited PTVs, including protein-truncating homozygous or compound heterozygous variants in autosomes (genes carrying one PTV and one Dmis in trans were also defined as biallelic PTVs); (2) biallelically inherited Dmis variants, including homozygous or compound heterozygous Dmis variants in autosomes; (3) biallelically inherited synonymous variants in autosomes; (4) X-linked inherited PTVs in males; (5) X-linked inherited Dmis variants in males, and (6) X-linked inherited synonymous variants in males. For each family, biallelically inherited variants in autosomes that were shared between the probands and siblings were removed, and only proband- or sibling-specific biallelically inherited variants were counted. Additionally, the X-linked inherited variants that were shared between male probands and male siblings were excluded. Fisher's exact test was used to calculate the statistical significance of differences in mutational burden between probands with ASD and unaffected siblings. Moreover, we limited the mutational burden analysis to brain-expressed genes and biallelically or hemizygous constrained genes. The brain-expressed genes were defined as genes with an average expression value ≥ 1 read per kilobase per million map reads [RPKM] in human fetal brain samples based on the BrainSpan database (https://www.brainspan.org/) [45]. The biallelically or hemizygous constrained genes were defined as genes carrying no more than five homozygous or hemizygous Dmis variants and no more than two homozygous or hemizygous PTVs in the gnomAD database.

### Prioritization of ASD-associated RIGs

Because biallelically inherited and X-linked inherited PTVs significantly differed between the probands and unaffected siblings, we focused on these PTVs to further prioritize candidate genes and filtered them, as the following standard: (1) PTVs recorded as benign variants in the ClinVar database; (2) X-linked inherited PTVs that were present in > 10 males in the gnomAD database; (3) genes in autosomes harboring biallelically inherited PTVs in the unaffected siblings or genes in the X chromosome harboring X-linked inherited PTVs in the unaffected male siblings; and (4) genes in the X chromosome with a probability of loss-of-function intolerance (pLI) < 0.5, as sourced from the gnomAD database. The phenotypes and inheritance patterns of the prioritized RIGs were curated using the Online Mendelian Inheritance in Man (OMIM: https://omim.org) database [46]. We applied the guidelines of the American College of Medical Genetics and Genomics [47] to interpret the clinical pathogenicity of inherited variants of known disease-associated genes, which were classified into pathogenic/likely pathogenic and variants of uncertain significance (VUS). Inherited variants of newly reported candidate genes were all interpreted as VUS. Additionally, the RIGs prioritized in this study were integrated with 49 RIGs from the ASC cohort [39] and other studies [36, 37] for further analysis.

A total of 87 DNGs with a false discovery rate (FDR) < 0.1 were sourced from our recently developed Gene4Denovo database (https://genemed.tech/gene4denovo) [48]. As described in our previous studies [29, 32], we used the RNA sequencing (RNA-seq) data of 524 human brain samples from 16 brain regions across different developmental stages from the BrainSpan database and protein–protein interaction (PPI) data from the STRING database (https://string-db.org/) [49] to evaluate functional relationships between ASD-associated RIGs and DNGs. Pearson’s correlation coefficients (*r*) between gene pairs were determined based on their expression in the human brain samples. When |*r*|≥ 0.8, genes were regarded as co-expressed, and if two proteins had a PPI confidence score ≥ 400, they were regarded to interact. A permutation test, as described in our previous studies [29, 32], was performed to evaluate the relationships between ASD-associated RIGs and DNGs while considering gene-level background DNV rates and gene lengths.

### Functional clusters of ASD-associated RIGs and DNGs

Co-expression and PPI data were used to construct a functional network of RIGs and DNGs. We paired any two ASD-associated RIGs or DNGs if they were co-expressed or found to interact at the protein level and combined these relationships to form an interconnected network, which was visualized in Cytoscape (v.3.6: https://cytoscape.org/). Gene ontology (GO) enrichment analysis of RIGs and DNGs was performed by using Metascape (https://metascape.org/) with default parameters. Similar GO terms were merged, and only the most significant GO term in each cluster was shown. Additionally, we sourced fragile X mental retardation protein (FMRP) targets [50] and essential genes [51], as described in a previous study [18].

### Brain expression patterns of RIGs and DNGs

RNA-seq data from the BrainSpan database were used to determine the spatiotemporal expression patterns of ASD-associated RIGs and DNGs, as described in previous studies [29, 32]. Hybrid-weighted gene co-expression network analysis (WGCNA) [52] was conducted to cluster ASD-associated RIGs and DNGs into different co-expression modules using standard protocols and at a power of four. Additionally, we sourced transcriptome data from different layers of the developing neocortex (*n* = 526) [53] and characterized the laminar expression patterns of ASD-associated RIGs and DNGs by using WGCNA at a power of three. Furthermore, we downloaded a single-cell RNA-seq dataset from 15,928 nuclei in the human middle temporal gyrus from the Allen Brain Map database (https://portal.brain-map.org/atlases-and-data/rnaseq). These transcriptional profiles showed the RNA levels of all genes in 45 types of inhibitory neurons and 24 types of excitatory neurons. For each gene in each nucleus, the RPKM value (i.e., mRNA expression) was calculated based on the counts per million value downloaded from the Allen Brain Map database. Subsequently, the average expression of each gene in each neuronal type was quantified. Differences in ASD gene expression in inhibitory and excitatory neurons were evaluated by the Wilcoxon-rank sum test.

## Results

### Excess biallelically inherited and X-linked inherited PTVs in patients with ASD

In this study, we analyzed biallelically inherited variants in autosomes and X-linked inherited variants in samples from males in 1799 quad families, including 1,799 probands (1571 males and 228 females), 1799 unaffected siblings (847 males and 952 females), and their unaffected parents. Because autosomal biallelically inherited variants shared between probands and their unaffected siblings are less likely to contribute to ASD etiology, they were removed from burden analysis, thus only proband- and sibling-specific inherited variants were included (Table 1). Moreover, X-linked inherited variants shared between male probands and unaffected males were also removed. As a result, 25 of 1799 probands and 19 of 1,799 siblings harbored biallelically inherited PTVs with no significant difference (*p* = 0.45, odds ratio (OR) = 1.32). Furthermore, there was no significant difference between the numbers of probands and siblings carrying biallelically inherited Dmis variants in the 1799 quads (*n* = 32 vs. 30, *p* = 0.89, OR = 1.07). However, significantly more male probands (55 of 1571) carried X-linked inherited PTVs than male siblings (15 of 847) (*p* = 0.015, OR = 2.01). Additionally, there was no significant difference in X-linked inherited Dmis variants between male probands and male siblings (*n* = 125 vs. 55, *p* = 0.22, OR = 1.24). Following negative control comparisons, we observed no significant enrichment in biallelically inherited synonymous variants in probands compared to that in siblings (*n* = 65 vs. 61, *p* = 0.79, OR = 1.07) or in X-linked inherited synonymous variants in male probands compared to male siblings (*n* = 392 vs. 202, *p* = 0.55, OR = 1.06; Table 1).

**Table 1:** Number of probands and unaffected siblings with autosomal biallelically or X-linked inherited variants

Group	Biallelic PTV (MAF ≤ 1%)	Biallelic Dmis (MAF ≤ 1%)	Biallelic Syn (MAF ≤ 1%)	X-linked PTV (MAF ≤ 0.1%)	X-linked Dmis (MAF ≤ 0.1%)	X-linked Syn (MAF ≤ 0.1%)
All genes						
Shared	8	7	2	7	24	93
ASD-specific	25	32	65	55	125	392
Sibling-specific	19	30	61	15	55	202
*P*-value	0.45	0.89	0.79	**0.015**	0.22	0.55
OR	1.32	1.07	1.07	2.01	1.24	1.06
95% Cl	0.70–2.55	0.63–1.83	0.74–1.55	1.11–3.86	0.89–1.76	0.87–1.30
Brain-expressed biallelically or hemizygous constrained genes						
Shared	2	3	0	1	15	63
ASD-specific	10	19	43	23	73	285
Sibling-specific	2	15	35	4	29	133
*P* value	**0.038**	0.61	0.42	**0.026**	0.17	0.14
OR	5.02	1.27	1.23	3.13	1.37	1.14
95% Cl	1.07–47.19	0.61–2.69	0.76–1.99	1.06–12.49	0.87–2.21	0.95–1.50

The rare recessive inherited coding variants are divided into biallelic variants (homozygous or compound heterozygous variants) in autosomes and X-linked hemizygous variants in males. “Shared” refers to the number of probands and siblings in each family who sharing a certain class of recessive inherited variants, and these were excluded from the burden analysis. X-linked inherited variants that were shared between a male proband and his male sibling were counted in the line of “Shared” and were excluded from the burden analysis. Biallelic variants were counted in 1799 probands and 1799 unaffected siblings. Inherited X-linked hemizygous variants were counted in 1571 male probands and 847 male siblings. Genes harboring a PTV and a Dmis variant in trans were included in the group of biallelic PTVs. Fisher's exact test was used to calculate *p* values between ASD probands and unaffected siblings. CI, confidence interval; Dmis, deleterious missense variants; OR, odds ratio; PTVs, protein-truncating variants, including stop-gain, stop-loss, splicing site variants, and frameshift indels; Syn, synonymous variants. *P* value below 0.05 was highlighted in bold

We then assessed inherited PTVs and Dmis variants in biallelically or hemizygous constrained genes (according to the gnomAD database) that were expressed in the brain (according to the BrainSpan database), and observed that probands harbored significantly more biallelically inherited PTVs in autosomes (*n* = 10 vs 2, *p* = 0.038, OR = 5.02) and X-linked inherited PTVs in males (*n* = 23 vs. 4, *p* = 0.026, OR = 3.13) relative to their siblings (Table 1). However, we did not observe significant differences in biallelically inherited Dmis variants (*n* = 19 vs. 15, *p* = 0.61, OR = 1.27) or X-linked inherited Dmis variants (n = 73 vs. 29, *p* = 0.17, OR = 1.37) between probands and siblings. These results suggest that biallelically and X-linked inherited PTVs contribute more significantly to ASD than do Dmis variants (Table 1).

### RIGs present higher genetic heterogeneity than DNGs in ASD

Based on the above analysis, we detected biallelically and X-linked inherited PTVs and Dmis variants in 33 genes in ASD probands. After filtering (Table S1), 21 ASD-associated RIGs were prioritized, including eight autosomal genes, and 13 X-linked genes (Fig. 1 and Table 2). Of these, 11 genes (*AFF2*, *ATAD3A*, *CCDC22*, *CUL4B*, *HDAC8*, *MED12*, *RBMX*, *RFT1*, *UBE2A*, *USP9X*, and *VPS13B*) have known links to neurodevelopmental disorders, two (*ANO5* and *CYBB*) are involved in other genetic disorders, and associations between the remaining eight genes and genetic disorders are uncertain according to the OMIM database. We noted that 14 probands with biallelically or X-linked inherited variants in the RIGs, including five patients with biallelic variants in autosomal genes and nine male patients with X-linked variants, had an intelligence quotient (IQ) ≤ 90. Four of the identified genes (*CUL4B*, *HDAC8*, *MED12,* and *USP9X*) have been previously reported to harbor de novo PTVs and Dmis variants in male cases with neurodevelopmental disorders (Table S2), providing more genetic evidence of their pathogenicity.

**Table 2:** Patients with autosomal biallelically or X-linked inherited PTVs or Dmis variants in the 21 prioritized candidate genes

Gene	Proband (gender)	Type	Location (hg19)	Ref	Alt	GenBank No	Functional effect	Nucleotide change	AA. alteration	gnomAD allele frequency	pLI	Phenotypes in OMIM (Inheritance)	ACMG
Autosomal genes (*n* = 8)													
* ANO5*^*#*^	14,640.p1 (M)	Chet	chr11:22,242,756	G	A	NM_213599	SP/Syn	c.294G > A	SP/p.A98A	0.0007	0	Gnathodiaphyseal dysplasia (AD); Miyoshi muscular dystrophy 3 (AR); Muscular dystrophy, limb-girdle, autosomal recessive 12 (AR)	VUS
			chr11: 22,294,441	C	G	NM_213599	Mis	c.2141C > G	p.T714S	0.0009			P
ATAD3A^*^	14,517.p1 (M)	Chet	chr1:1,469,361	G	A	NM_001170535	Mis	c.1670G > A	p.R557H	0.0002	0	Harel-Yoon syndrome (AD, AR); Pontocerebellar hypoplasia, hypotonia, and respiratory insufficiency syndrome, neonatal lethal (AR)	VUS
			chr1:1,469,380	G	-	NM_001170535	FS	c.1689delG	p.Q563Hfs*9	0			P
* ATG7*	13,998.p1 (M)	Chet	chr3:11,389,351	G	T	NM_006395	SP/Mis	c.1126G > T	SP/p.G376C	5.58E-05	0		VUS
			chr3:11,404,365	G	A	NM_006395	Mis	c.1762G > A	p.V588M	0.0001			VUS
* HIPK3*	13,076.p1 (M)	Homo	chr11:33,374,639	T	C	NM_001048200	SP/Mis	c.3110 T > C	SP/p.V1037A	0.0008	0.28		VUS
*I NTS4*	14,051.p1 (M)	Chet	chr11:77,635,920	G	A	NM_033547	SG	c.1390C > T	p.R464X	1.23E-05	0		VUS
			chr11:77,649,828	G	A	NM_033547	Mis	c.1034C > T	p.S345L	0.0004			VUS
* LLGL1*	14,404.p1 (M)	Homo	chr17:18,138,154	G	A	NM_004140	SP/Mis	c.907G > A	SP/p.G303S	0.0009	0		VUS
RFT1*	11,176.p1 (M)	Chet	chr3:53,133,472	T	C	NM_052859	Mis	c.1133A > G	p.Y378C	0.0005	0	Congenital disorder of glycosylation (AR)	VUS
			chr3:53,126,384	C	–	NM_052859	SP	c.1458 + 1G > -	SP	4.53E-06			P
VPS13B^*^	12,651.p1 (M)	Chet	chr8:100,829,780	G	A	NM_017890	Mis	c.8185G > A	p.G2729R	7.98E-06	0	Cohen syndrome (AR)	LP
			chr8:100,396,500	G	A	NM_017890	SG	c.2889G > A	p.W963X	0			P
X-linked genes (*n* = 13)													
AFF2^*^	14,096.p1 (M)	Hem	chrX:148,068,931	-	C	NM_001170628	FS	c.2582dupC	p.I863Hfs*6	0	1	Mental retardation, X-linked, FRAXE type (XLR)	P
* APOO*	14,187.p1 (M)	Hem	chrX:23,886,804	G	A	NM_024122	SP/Syn	c.294C > T	SP/p.D98D	0	0.6		VUS
CCDC22^*^	12,325.p1 (M)	Hem	chrX:49,105,278	A	C	NM_014008	SP/Mis	c.1432A > C	SP/p.M478L	0	1	Ritscher-Schinzel syndrome 2 (XLR)	LP
	11,720.p1 (M)		chrX:49,105,359	C	T	NM_014008	Mis	c.1513C > T	p.R505W	6.18E-05			LP
CUL4B^*^	14,363.p1 (M)	Hem	chrX:119,708,407	A	G	NM_003588	SP/Syn	c.66 T > C	SP/p.G22G	0	1	Mental retardation, syndromic 15 (XLR)	LP
* CYBB*#	14,070.p1 (M)	Hem	chrX:37,664,420	A	G	NM_000397	SP/Mis	c.1313A > G	SP/p.K438R	4.39E-05	1	Chronic granulomatous disease, X linked (XLR); Immunodeficiency 34, mycobacteriosis (XLR)	LP
* GRIPAP1*	14,524.p1 (M)	Hem	chrX:48,837,828	C	T	NM_020137	SP/Syn	c.1830G > A	SP/p.A610A	0.0001	1		VUS
HDAC8^*^	13,136.p1 (M)	Hem	chrX:71,694,562	G	-	NM_001166419	FS	c.755delC	p.P252Qfs	7.35E-05	0.98	Cornelia de Lange syndrome 5 (XLD)	LP
* IL13RA1*	11,167.p1 (M)	Hem	chrX:117,881,009	T	G	NM_001560	Mis	c.321 T > G	p.S107R	0	0.96		VUS
	11,488.p1 (M)		chrX:117,895,252	C	T	NM_001560	SP/Syn	c.828C > T	SP/p.Y276Y	2.48E-05			VUS
MED12^*^	11,217.p1 (M)	Hem	chrX:70,343,445	G	A	NM_005120	SP/Mis	c.1619G > A	SP/p.R540H	1.33E-05	1	Lujan-Fryns syndrome (XLR), Ohdo syndrome(XLR), Opitz-Kaveggia syndrome (XLR)	VUS
	12,626.p1 (M)		chrX:70,352,298	A	G	NM_005120	Mis	c.4325A > G	p.H1442R	0			LP
RBMX^*^	13,063.p1 (M)	Hem	chrX:135,957,417	C	T	NM_002139	SP/Mis	c.782G > A	SP/p.S261N	0	0.83	Mental retardation, X-linked, syndromic 11, Shashi type (XLR)	LP
* SLC38A5*	14,423.p1 (M)	Hem	chrX:48,325,258	C	T	NM_033518	SP/Mis	c.247G > A	SP/p.A83T	0	0.98		VUS
UBE2A^*^	12,440.p1 (M)	Hem	chrX:118,716,638	A	T	NM_003336	SP/Mis	c.329A > T	SP/p.Q110L	0	0.82	Mental retardation, X-linked syndromic, Nascimento-type (XLR)	LP
USP9X^*^	13,929.p1 (M)	Hem	chrX:40,982,891	A	G	NM_001039590	Mis	c.10A > G	p.T4A	1.52E-05	1	Mental retardation, X-linked 99(XLR); Mental retardation, X-linked 99, syndromic, female-restricted (XLD)	LP
	11,358.p1 (M)		chrX:40,996,058	G	A	NM_001039590	SP/Mis	c.437G > A	SP/p.R146K	0			LP
	12,628.p1 (M)		chrX:41,012,318	G	C	NM_001039590	Mis	c.1881G > C	p.M627I	1.39E-05			LP

Genes marked with * are implicated in neurodevelopmental disorders, and those marked with # are associated with other genetic disorders. The coding variants within ± 2 bp of the exon–intron boundary were defined as cryptic splice sites using ANNOVAR. All X-linked hemizygous inherited variants were analyzed in males. The reference sequence of a certain gene in the ClinVar database or the transcript with the highest expression in the human brain based on the GTEx database was selected to annotate the recessive inherited variants. AD, autosomal dominant; AR, autosomal recessive; Chet, compound heterozygous; Dmis, deleterious missense; F, female; FS, frameshift; Hem, hemizygous; Homo, homozygous; LP, likely pathogenic; M, male; Mis, missense; P, pathogenic; pLI, probability of loss-of-function intolerance; PTVs, protein-truncating variants; SG, stop-gain; SP, splicing; Syn, synonymous; XLD, X-linked dominant; XLR, X-linked recessive; VUS, variant of uncertain significance. The variants in known disease genes were interpreted according to ACMG guidelines and other variants were determined as VUSs. The variants in *VPS13B* were reported in a previous study [37]

Proband 12,651.p1, with an IQ of 34, carried pathogenic/likely pathogenic compound heterozygous variants (c.8185G > A, p.G2729R; c.2889G > A, p.W963X) in *VPS13B,* and was previously confirmed to have Cohen syndrome [37]. Proband 12,440.p1 with an IQ of 58, carried a likely pathogenic X-linked hemizygous splicing variant in *UBE2A* (c.329A > T, p.Q110L, at the end of exon 5), a known causative gene of ID. Among the eight newly reported genes, two are involved in the nervous system. First, we identified an X-linked inherited variant in *GRIPAP1* at the end of exon 20 (c.1830G > A, p.A610A), which might alter RNA splicing. *GRIPAP1* is specifically expressed in the nervous system and encodes a neuron-specific guanine nucleotide exchange factor for the Ras family of small G proteins [54]. Moreover, *GRIPAP1* might regulate α-amino-3-hydroxy-5-methyl-4-isoxazolepropionic acid receptor location and synaptic signal transmission in the brain [54]. Besides, a recent study identified an X-linked variant of apolipoprotein (*APOO*: c.350 T > c, p.I117T) among members of a family with mitochondrial disease, and most affected individuals exhibited developmental delay, cognitive impairment, and autistic features [55]. Here, we identified a potential inherited splicing variant of *APOO* that was not present in the gnomAD database.

The 21 RIGs harbored biallelically or X-linked inherited PTVs or Dmis variants in 26 of 1,799 (1.4%) patients in the SSC cohort (Table 2). X-linked inherited variants in three genes (*IL13RA1, MED12,* and *CCDC22*) were detected in two unrelated probands, and X-linked inherited variants in *USP9X* were detected in three unrelated probands. Additionally, 41 autosomal ASD-associated RIGs identified in a previous study based on the ASC cohort [39] were present in 44 of 2343 (1.88%) patients; these RIGs included three genes (*CDHR3*, *FEV*, and *PAH*) with variants in two unrelated patients and the others only in one patient (Table S3). No overlap was observed between the 41 autosomal RIGs from the ASC cohort and eight autosomal RIGs from the SSC cohort, suggesting high genetic heterogeneity among ASD-associated RIGs. Based on the genetic data regarding probands in the SSC cohort, we found that four of the 21 RIGs harbored multiple recessive inherited variants, whereas 36 of 87 DNGs with FDR < 0.1 from the Gene4Denovo database (Table S4) harbored multiple DNVs with slight differences (Fisher’s exact test: *p* = 0.078, OR = 0.34), indicating a higher genetic heterogeneity in RIGs than DNGs in ASD.

### RIGs have higher functional heterogeneity than DNGs in ASD

After combining the 21 RIGs prioritized in this study with 41 from the ASC cohort [39] and 10 from two other previous studies [36, 37], 70 RIGs were included for further analysis after removing redundancy (Table S3). We investigated associations between RIGs and DNGs using transcription data from the BrainSpan database and PPI data from the STRING database. RIGs were co-expressed with 16 DNGs (*p* = 0.045) and formed 30 connections (*p* = 0.043), with the frequency of co-expression and connections significantly higher than randomly expected (Figure S1a). Additionally, we observed that ASD-associated RIGs interacted with 14 DNGs (*p* = 0.042) and 23 connections (*p* = 0.015) at the protein level based on PPI data, which was also significantly higher than those randomly expected (Figure S1b), again suggesting that ASD-associated RIGs were functionally related to DNGs.

**Fig. 2:**
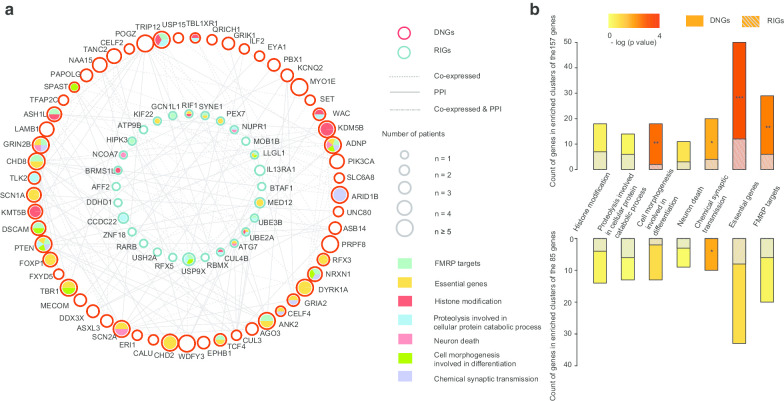
Functional network of ASD-associated RIGs and DNGs. **a** Network of ASD-associated RIGs and DNGs based on PPI and co-expression in the human brain. **b** Comparison of the numbers of RIGs and DNGs in the functional clusters. There were 85 genes in the network and 157 genes (RIGs and DNGs) in total. *p ≤ 0.05; **p ≤ 0.01; ***p ≤ 0.001. ASD, autism spectrum disorder; RIGs, recessive inherited genes; DNGs, genes with de novo variants; PPI, protein–protein interaction

We then connected any RIG or DNG that was co-expressed in the human brain at the transcript level or via interaction at the protein level to develop a functional network, that comprised 58 DNGs and 37 RIGs (Fig. 2a). This network was enriched in genes involved in histone modification (GO: 0016570, *p* = 1.07 10^–9^), proteolysis involved in cellular protein catabolic process (GO: 0051603, *p* = 2.76 10^–4^), neuron death (GO: 0070997, *p* = 2.02 10^–5^), cell morphogenesis involved in differentiation (GO: 0000904, *p* = 1.96 10^–6^) and chemical synaptic transmission (GO: 0007268, *p* = 4.30 10^–8^) (Fig. 2b and Table S5). Additionally, we observed significant enrichment of FMRP targets (*p* = 2.96 10^–11^) and essential genes (*p* = 2.69 10^–9^) in this functional network. We found that DNGs were more likely to be associated with chemical synaptic transmission and essential genes (*p* < 0.05) than RIGs (Fig. 2b). Moreover, functional analysis of all 157 genes (70 RIGs and 87 DNGs) indicated that DNGs were more likely to be associated with chemical synaptic transmission and cell morphogenesis involved in differentiation, FMRP targets, and essential genes than RIGs (all *p* < 0.05) (Fig. 2b). The same analysis was performed between the 70 RIGs and 102 ASD-associated DNGs recently identified by Satterstrom et al. [56] with similar results observed (Figure S2). These findings indicated that RIGs were less likely to be enriched in functional clusters involved in ASD etiology and might present higher functional heterogeneity than DNGs.

### RIGs have higher heterogeneity than DNGs in brain expression patterns

**Fig. 3:**
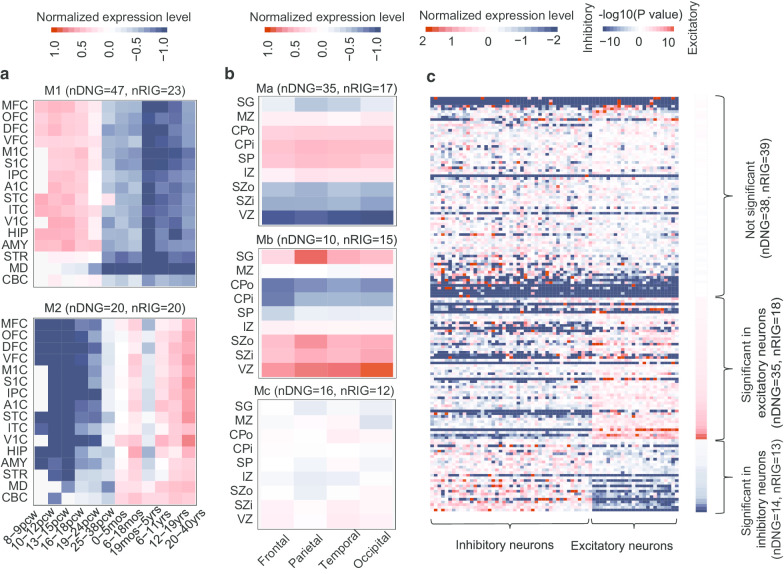
Expression patterns of ASD-associated RIGs and DNGs in the human brain. **a** Heat map of the normalized expression levels of two spatiotemporal co-expression modules (M1 and M2), corresponding to 17 developmental stages and 16 sub-regions in the human brain. pcw, post-conceptual weeks; yrs, years. CBC, cerebellum cortex; MFC, anterior cingulate cortex; OFC, orbital frontal cortex; DFC, dorsolateral prefrontal cortex; VFC, ventrolateral prefrontal cortex; M1C, primary motor cortex; S1C, primary somatosensory cortex; IPC, posteroinferior parietal cortex; A1C, primary auditory cortex; STC, posterior superior cortex; ITC, inferolateral temporal cortex; VIC, primary visual cortex; HIP, hippocampus; AMY, amygdaloid complex; STR, striatum; MD, mediodorsal nucleus of thalamus; CBC, cerebellar cortex. **b** Heat map of the normalized expression levels of all RIGs and DNGs in different cortical layers. SG, subpial granular zone; MZ, marginal zone; CPo, outer cortical plate; CPi, inner cortical plate; SP, subplate zone; IZ, intermediate zone; SZo, outer subventricular zone; SZi, inner subventricular zone; VZ, ventricular zone. (C) Heat map of the normalized expression levels of RIGs and DNGs in 45 inhibitory and 24 excitatory neuronal types. *P* values were calculated by Wilcoxon rank sum test. Expression level is presented according to the log^2^ value of the average expression of each gene

To explore the spatiotemporal expression patterns of RIGs and DNGs in the human brain, we performed WGCNA of all samples at different developmental stages from the BrainSpan database to identify co-expression modules. We identified two independent modules with different spatiotemporal expression patterns, comprising a total of 43 (61.43%) of 70 RIGs and 67 (77.01%) of the 87 DNGs (Fig. 3a and Table S6). Genes in module 1 (M1: *n* = 70) were highly expressed during the prenatal stages, but gradually decreased toward the end of the prenatal stage and remained relatively stable after birth. In contrast, genes in module 2 (M2: *n* = 40) exhibited the opposite spatiotemporal expression pattern. Although RIGs presented a spatiotemporal expression pattern similar to that of DNGs, as described in our previous study [29] and others studies [57, 58], we found that DNGs were more likely to be associated with M1 than RIGs (*n* = 47 vs. 23, Fisher’s test, *p* = 0.022, OR = 2.19) (Table S7).

Because the expression of RIGs and DNGs showed significant fluctuation in the human brain during the prenatal period, we characterized prenatal neocortical-expression patterns using previously reported laser-microdissection data from prenatal neocortical samples [53]. Interestingly, we identified three co-expression modules (Ma, Mb, and Mc) with distinct laminar expression patterns, and comprising 44 (62.86%) RIGs and 61 (70.11%) DNGs (Fig. 3b and Table S6). The expression of genes within Ma (*n* = 52), the biggest module, was higher in the middle layers (from the marginal zone to subplate zone) and lower in the deep cortical layers and the outer subventricular zone to the ventricular zone. In contrast, gene expression in Mb (*n* = 25) displayed the opposite trends. However, genes within Mc (*n* = 31) did not display any laminar expression features. Although the three modules contained both RIGs and DNGs, we found that DNGs were significantly more likely to be associated with Ma than RIGs (*n* = 35 vs. 17, *p* = 0.041, OR = 2.09), whereas RIGs were more likely to be associated with Mb than DNGs (*n* = 10 vs. 15, *p* = 0.12, OR = 0.48).

Complex neuronal diversity and connectivity are vital to human brain function [59]. Single-nucleus RNA-seq analysis of the human brain allows the characterization of the inhibitory and excitatory neuronal expression patterns of ASD-associated RIGs. Therefore, we compared the expression of RIGs and DNGs in 45 types of inhibitory (GABAergic) neurons and 24 types of excitatory (glutamatergic) neurons. In total, 53 genes (nRIG = 18 and nDNG = 35) showed higher expression in excitatory neurons than in inhibitory neurons, whereas 27 genes (nRIG = 13 and nDNG = 14) showed higher expression in inhibitory neurons than in excitatory neurons, with 77 genes exhibiting no significant difference between neuron classes (Fig. 3c and Table S7). Although 50% of genes were broadly expressed in both inhibitory and excitatory neurons, more genes containing RIGs and DNGs were preferentially expressed in excitatory neurons, thereby highlighting their essential roles in the cortical circuit. Notably, although the difference was not significant, DNGs were more likely to be associated with excitatory neurons than RIGs (*p* = 0.063, OR = 1.94). Taken together, these results revealed that RIGs were less likely to present distinct expression patterns in the human brain, suggesting higher transcriptomic heterogeneity than DNGs.

To validate whether the observations between RIGs and DNGs were ASD specific, we extracted ID-associated RIGs (*n* = 120) from the OMIM database and DNGs (*n* = 82) from the Gene4Denovo database. ID-associated DNGs tended to be associated with the module that was highly expressed during the prenatal stages (M1: OR = 1.64, *p* = 0.088) (Table S7), and were more likely to be associated with excitatory neurons than RIGs (OR = 2.06, *p* = 0.040) (Table S7). We then compared the expression patterns of congenital heart disease (CHD) associated RIGs (*n* = 88) and DNGs (*n* = 78) from a previous study [60] and the Gene4Denovo database, respectively, and did not observe a significant enrichment of DNGs relative to RIGs in M1 (OR = 1.22, *p* = 0.53) or in the middle cortical layers (Ma: OR = 1.04, *p* = 1.0). Additionally, compared with RIGs, DNGs associated with CHD were not more enriched in excitatory neurons (OR = 0.98, *p* = 1.0) (Table S7). These observations suggest that the differences in expression patterns between DNGs and RIGs are specific to neurodevelopmental disorders.

## Discussion

ASD is highly heritable, and DNVs are estimated to contribute to the disease in up to 30% of cases; however, the genetic defects in most ASD cases remain unclear [61]. Few studies have attempted to evaluate the effects of inherited PTVs and prioritize ASD-associated RIGs [36–39]. In 1799 quads from the SSC cohort, we observed that ASD probands were more likely to carry biallelically and X-linked inherited PTVs in brain-expressed and biallelically or hemizygous constrained genes than unaffected siblings, consistent with a previous study [38]. Given that a trend toward the enrichment of recessive inherited Dmis variants has been observed in ASD [39], we believe that a fraction of these variants might be involved in disease etiology. However, larger sample sizes and functional studies will be required to estimate the contributions of these variants to ASD.

We prioritized 21 RIGs with biallelically or X-linked inherited PTVs and Dmis variants found in ASD probands only, including 13 genes associated with known genetic disorders. We hypothesized that these ASD-associated RIGs contribute to a broad range of neurological phenotypes, with different penetrance in each phenotype. Furthermore, we reported eight genes yet to be linked to any genetic disorders, with possible associations with ASD. The biallelically or X-linked inherited variants of the 21 RIGs were present in 1.4% of ASD cases in the SSC cohort, which was close to estimations (1.8–3%) in previous large cohort studies [38, 39]. We encourage further studies to validate our candidate genes, detect more inherited variants in neurodevelopmental disorders, and characterize genotype–phenotype correlations, which would contribute to ASD subtype definitions.

Although a previous study detected four RIGs in ASD [36], none of them harbored biallelically or X-linked inherited variants in the ASC cohort or in this study. Yu et al. [37] detected six RIGs, one of which (*PAH*) was found to harbor different biallelically inherited variants in the ASC cohort (Table S3). Similarly, Doan et al. [39] reported 41 RIGs in 2343 probands, and identified only three genes with variants in more than one proband. In the present study, we identified 21 RIGs from 1799 quads and found four X-linked genes harboring inherited variants in multiple probands. Most RIGs harbored biallelically or X-linked inherited variants in only one proband, in both the SSC and ASC cohorts, with few RIGs overlapping between cohorts; this highlighted the high genetic heterogeneity of ASD-associated RIGs and the need for larger sample sizes in future studies. However, we cannot fully exclude the influence of technical differences between different studies.

This study highlights the genetic similarities and differences between ASD-associated RIGs and DNGs from different perspectives. First, although both DNGs and RIGs displayed genetic heterogeneity, RIGs were less likely to harbor multiple biallelically or X-linked inherited variants than DNGs were to carry multiple DNVs, suggesting a higher genetic heterogeneity in RIGs. Second, RIGs and DNGs were functionally interconnected, forming a functional network of known pathways involved in ASD [13]. However, some functional clusters showed a preference for DNGs, whereas no functional cluster showed a preference for RIGs, further suggesting higher functional heterogeneity in RIGs. Third, RIGs were significantly co-expressed with DNGs at the mRNA level in the human brain and shared spatiotemporal expression patterns, neocortex laminar expression patterns, and excitatory neuronal expression patterns with DNGs [29, 62]. However, RIGs were less likely to be associated with prenatal brain regions, middle cortical layers, and excitatory neurons, which are associated with ASD and other neuropsychiatric disorders [29, 34, 62–64], again suggesting the higher transcriptomic heterogeneity of RIGs. Furthermore, expression patterns of RIGs and DNGs were similar in ASD and ID cases but differed from those in CHD cases, suggesting that these expression patterns might be specific to neurodevelopmental disorders.

### Limitations

We acknowledge several limitations in this study. First, because we only observed significant differences in biallelically and X-linked inherited PTVs between probands and siblings rather than Dmis variants, we prioritized candidate genes with PTVs and might have missed promising candidate genes harboring Dmis variants. Second, the 21 candidate genes and biallelically or X-linked inherited variants should be confirmed in additional studies and require functional validation. Third, because the samples were derived from the SSC cohort, we were unable to re-examine patients with biallelically or X-linked inherited variants to further characterize their detailed clinical phenotypes.

## Conclusions

In summary, our study showed that biallelically or X-linked inherited variants contribute to ASD but only occur in only a small fraction of ASD cases. Moreover, the analyses of functional clusters and expression patterns suggested that ASD-associated RIGs were functionally correlated with DNGs; however, they presented higher genetic and functional heterogeneity, providing strong evidence for the need for further studies regarding the molecular etiology of ASD.

## Supplementary information

**Additional file 1:** **Figure S1**. Permutation test of connections between RIGs and DNGs. **Figure S2**. The functional network of 70 RIGs in this study and 102 DNGs from Satterstrom et al. **Table S1**. Summary of genes excluded when prioritizing candidate genes.**Table S2**. De novo variants in genes with X-linked inherited variants based on the Gene4Denovo database. **Table S3**. All RIGs in ASD included in this study. **Table S4**. ASD-associated DNGs were sourced from the Gene4denovo database. **Table S5**. Functional enrichment of ASD-associated RIGs and DNGs. **Table S6**. Expression patterns of 70 RIGs and 87 DNGs in ASD. **Table S7**. Comparison of expression patterns between DNGs and RIGs in ASD, ID and CHD.

## Data Availability

The data used in this study are available from the corresponding author upon reasonable request.

## Web Resources

Allen Brian Map, https://portal.brain-map.org/atlases-and-data/rnaseq

BrainSpan, https://www.brainspan.org

Cytoscape, https://cytoscape.org/

Gene4Denovo, http://genemed.tech/gene4denovo

gnomAD, https://gnomad.broadinstitute.org/

Metascape, https://metascape.org/

OMIM, https://omim.org

SFARI Base, https://www.sfari.org/resource/sfari-base/

STRING, https://string-db.org〹

## Abbreviations

ASC: Autism sequencing consortium
ASD: Autism spectrum disorder
CHD: Congenital heart disease
Dmis: Deleterious missense
DNGs: Genes with de novo variants
DNV: De novo variant
FDR: False discovery rate
FMRP: Fragile X mental retardation protein
gnomAD: Genome aggregation database
GO: Gene ontology
ID: Intellectual disability
IQ: Intelligence quotients
MAF: Minor allele frequency
OMIM: Online Mendelian Inheritance in Man
OR: Odds ratio
PPI: Protein–protein interaction
PTVs: Protein truncating variants
RIGs: Recessive inherited genes
RNA-seq: RNA sequencing
RPKM: Reads per kilobase per million
SSC: Simons simplex collection
VUS: Variants of uncertain significance
WES: Whole-exome sequencing
WGCNA: Weighted gene co-expression network analysis
