# Supplementary material for: Functional relationships between recessive inherited genes and genes with de novo variants in autism spectrum disorder
Source: Mol Autism. 2020 Oct 6;11:75. doi: 10.1186/s13229-020-00382-x (PMC7541261; doi:10.1186/s13229-020-00382-x)
Supplement: Supplementary file 1 — Additional file 1: Figure S1. Permutation test of connections between RIGs and DNGs. Figure S2. The functional network of 70 RIGs in this study and 102 DNGs from Satterstrom et al. Table S1. Summary of genes excluded when prioritizing candidate genes.Table S2. De novo variants in genes with X-linked inherited variants based on the Gene4Denovo database. Table S3. All RIGs in ASD included in this study. Table S4. ASD-associated DNGs were sourced from the Gene4denovo database. Table S5. Functional enrichment of ASD-associated RIGs and DNGs. Table S6. Expression patterns of 70 RIGs and 87 DNGs in ASD. Table S7. Comparison of expression patterns between DNGs and RIGs in ASD, ID and CHD. [file 13229_2020_382_MOESM1_ESM.pdf]

SUPPLEMENTARY MATERIALS

This file includes:

Figure S1. Permutation test of connections between RIGs and DNGs.

Figure S2. The functional network of 70 RIGs in this study and 102 DNGs from Satterstrom et al.

Table S1. Summary of genes excluded when prioritizing candidate genes.

Table S2. *De novo* variants in genes with X-linked inherited variants based on the Gene4Denovo database.

Table S3. All RIGs in ASD included in this study.

Table S4. ASD-associated DNGs were sourced from the Gene4denovo database.

Table S5. Functional enrichment of ASD-associated RIGs and DNGs.

Table S6. Expression patterns of 70 RIGs and 87 DNGs in ASD.

Table S7. Comparison of expression patterns between DNGs and RIGs in ASD, ID and CHD.

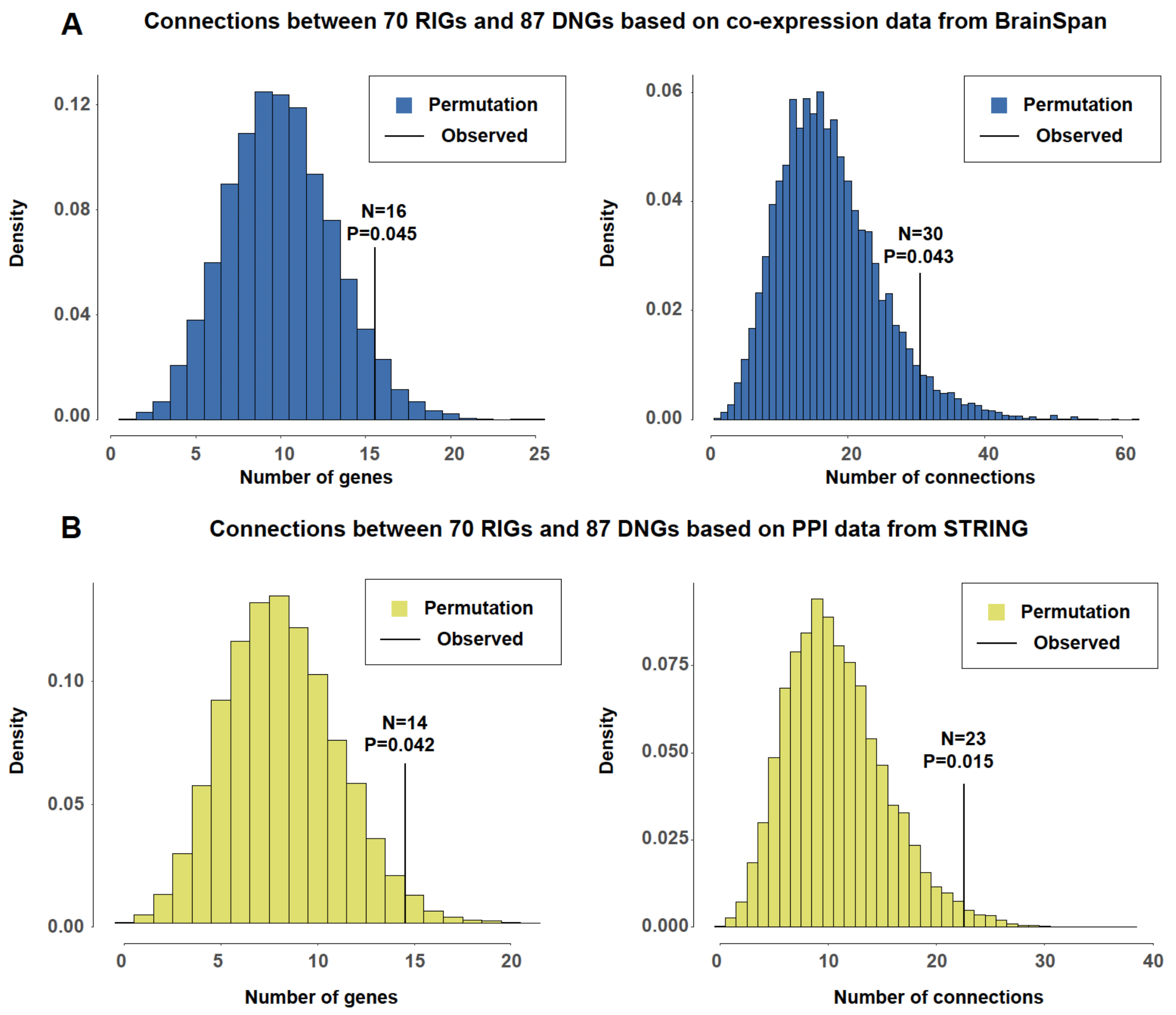

Figure S1. Permutation test of connections between RIGs and DNGs.

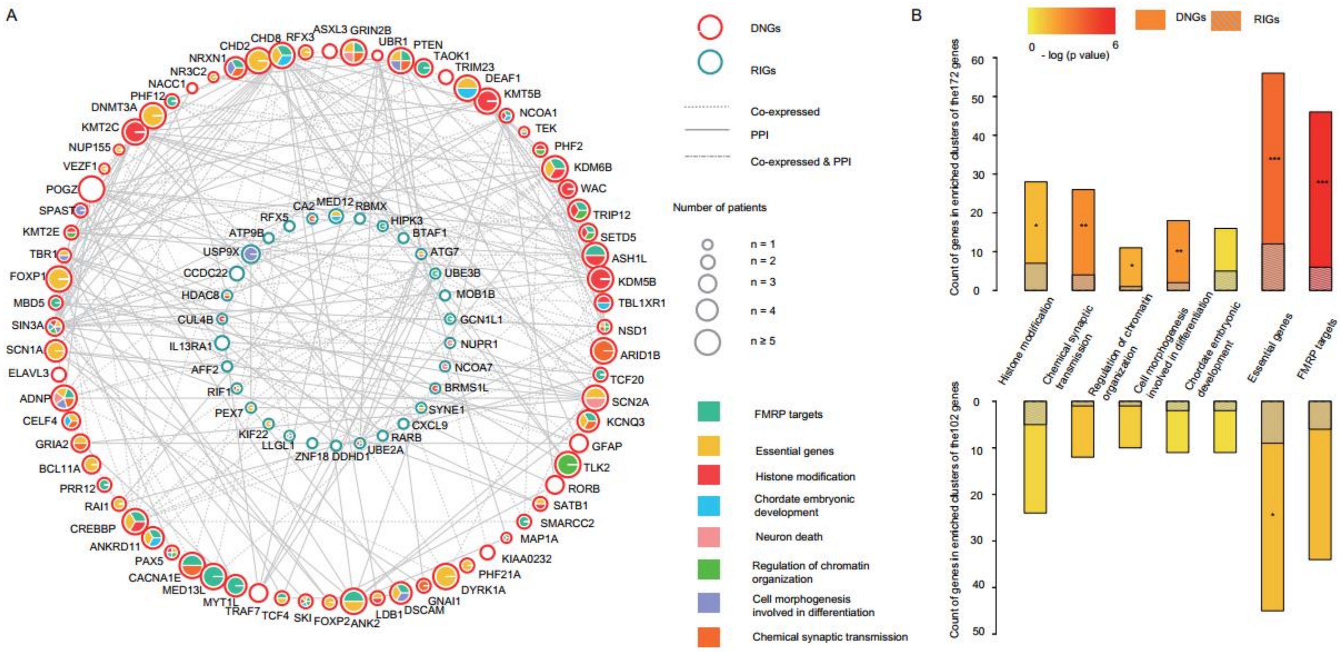

Figure S2. The functional network of 70 RIGs in this study and 102 DNGs from Satterstrom et al.

Table S1. Summary of genes excluded when prioritizing candidate genes.

Gene	Proband	Type	Location (hg19)	Ref	Alt	GenBank No.	Functional effect	Nucleotide change	AA. alteration	MAF in gnomAD	No. of males in gnomAD (*n*)	pLI	Exclusion reasons
DYNLT3	13311.p1	Hem	chrX:37700357	C	G	NM_006520	SP/Syn	c.198G>C	SP/p.V66V	0.0008	60	0.72	II
FMR1	12390.p1	Hem	chrX:147019617	G	A	NM_002024	SP	c.1126-1G>A	SP	0.001	94	0.65	II
HECW2	11564.p1	Homo	chr2:197208381	G	A	NM_001348768	SP/Mis	c.400C>T	SP/p.P134S	0.0013	-	1	I
LAGE3	14152.p1	Hem	chrX:153706622	A	G	NM_006014	SP/Mis	c.317T>C	SP/p.V106A	1.65E-05	0	0.13	IV
LRCH2	12528.p1	Hem	chrX:114399968	C	T	NM_020871	SP/Mis	c.1355G>A	SP/p.R452K	0.0004	14	1	II
MAP3K15	14181.p1	Hem	chrX:19389462	C	A	NM_001001671	SP	c.3294+1G>T	SP	0.00002	1	0	III
NXT2	12851.p1	Hem	chrX:108779205	A	G	NM_018698	SP/Mis	c.94A>G	SP/p.S32G	0.0004	23	0.83	II
PPP1R3F	12014.p1	Hem	chrX:49142296	G	C	NM_033215	SP/Mis	c.1144G>C	SP/p.V382L	7.38E-06	0	0	IV
SH3BP2	14614.p1	Chet	chr4:2824764	G	A	NM_001122681	SP/Mis	c.239G>A	SP/p.R80Q	0.0004	-	0	I
			chr4:2833298	G	A	NM_001122681	SP/Mis	c.1242G>C	SP/p.Q414H	0	-	0	-
VMA21	14018.p1	Hem	chrX:150573389	C	T	NM_001017980	SP/Syn	c.165C>T	SP/p.G55G	0.001	75	0.57	II
ZNF185	12373.p1	Hem	chrX:152101415	A	C	NM_001178106	SP	c.1020-2A>C	SP	0.0001	8	0	IV
ZNF41	12892.p1	Hem	chrX:47315748	C	T	NM_001324152	SG	c.224G>A	p.W75X	8.17E-05	1	0.04	IV

Chet, compound heterozygous; Homo, Homozygous; Hem, hemizygous; Mis, missense; SP, splicing; FS, frameshift; Syn, synonymous; SG, stop-gain. pLI, probability of loss-of-function intolerance. The exclusion strategies used to prioritize candidate genes were as follows: (I) PTVs recorded as benign variants in the ClinVar database; (II) X-linked inherited PTVs that were present in >10 males in the gnomAD database; (III) genes that harboured biallelically inherited PTVs in the unaffected siblings or harboured X-linked inherited PTVs in male siblings; and (IV) genes in the X chromosome that have a probability of loss-of-function intolerance (pLI) < 0.5, as sourced from the gnomAD database.

Table S2. *De novo* variants in genes with X-linked inherited variants based on the Gene4Denovo database.

Gene	Location (hg19)	Ref	Alt	Effects	Amino acid change	PMID	Disease
CUL4B	119669710	CA	-	frameshift	c.2134_2135del, p.L714Kfs*4	28135719	UDD [1]
HDAC8	71684459	C	T	missense	c.587G>A, p.C196Y	28135719	UDD [1]
HDAC8	71715024	C	T	missense	c.259G>A, p.D87N	28135719	UDD [1]
HDAC8	71684408	C	A	splicing	c.637+1G>T	28135719	UDD [1]
HDAC8	71684432	A	T	stopgain	c.614T>A, p.L205X	28135719	UDD [1]
HDAC8	71715077	A	G	missense	c.206T>C, p.L69P	28135719	UDD [1]
HDAC8	71710788	A	G	missense	exon4:c.346T>C:p.F116L	28135719	UDD [1]
HDAC8	71788721	T	A	stopgain	c.178A>T, p.K60X	28135719	UDD [1]
HDAC8	71684483	A	C	missense	c.563T>G, p.M188R	28135719	UDD [1]
MED12	70342357	G	C	splicing	c.1249-1G>C	27479843	ID [2]
MED12	70342356	A	G	splicing	c.1249-2A>G	27479843	ID [2]
MED12	70349949	T	A	missense	c.3932T>A, p.V1311E	28135719	UDD [1]
MED12	70349262	A	G	missense	c.3674A>G, p.K1225R	28135719	UDD [1]
MED12	70346296	T	C	missense	c.2647T>C, p.S883P	28135719	UDD [1]
MED12	70357668	C	A	stopgain	c.5919C>A, p.Y1973X	28135719	UDD [1]
USP9X	41025217	A	T	missense	c.2078A>T, p.D693V	25363768	ASD [3]
USP9X	41047364	T	A	stopgain	c.3804T>A, p.Y1268X	27479843	ID [2]
USP9X	41031089	A	G	splicing	c.3028-2A>G	27479843	ID [2]
USP9X	41047323	C	T	stopgain	c.3763C>T, p.Q1255X	28135719	UDD [1]
USP9X	41000634	C	T	stopgain	c.1111C>T, p.R371X	28135719	UDD [1]
USP9X	41029317	-	TG	frameshift insertion	c.2706_2707insTG, p.E903Wfs*25	28135719	UDD [1]
USP9X	41000679	A	-	frameshift deletion	c.1156delA, p.M386Wfs*12	28135719	UDD [1]
USP9X	41055613	-	GG	splicing	c.4086+1->GG	28135719	UDD [1]
USP9X	41069824	T	G	missense	c.5078T>G, p.L1693W	28135719	UDD [1]
USP9X	41027299	C	T	missense	c.2464C>T, p.R822C	28135719	UDD [1]

ASD, autism spectrum disorder; ID, intellectual disability; UDD, unspecified neurodevelopmental disorder. The protein truncating variants and deleterious missense variants in X-linked genes were sourced from the Gene4Denovo database [4] (with FDR < 0.1) (http:/genemed.tech/gene4denovo/).

Table S3. All RIGs in ASD included in this study.

Resource	Recessive inherited genes in ASD
ASC:<br>Ryan N. Doan, et al. Nature Genetics 2019 [5] (*n* = 41)	*ABCC10, ATP9B, BRMS1L, BTAF1, CA2, CDHR3, CGN, CHTF18, COL19A1, CPA4, CXCL9, DDHD1, DNAI2, DYDC1, ELOF1, EML5, FCHSD2, FEV, GCN1L1, GZF1, HOXC5, IFITM5, ITPRIPL1, KIF22, LSMEM1, MEDAG, MOB1B, NCOA7, NSUN2, NUPR1,PAH, RARB, RFX5, RIF1, ROGD1, SCGB1D1, SLC1A1, SLC22A6, SLC35E3, USH2A, ZNF16*
SSC:this study (*n* = 21)	*AFF2, ANO5, APOO, ATAD3A, ATG7, CCDC22, CUL4B, CYBB, GRIPAP1, HDAC8, HIPK3, IL13RA1, INTS4, LLGL1, MED12, RBMX, RFT1, SLC38A5, UBE2A, USP9X, VPS13B*
Maria H. Chahrour, et al. PLoS Genetics 2012 [6] (*n* = 4)	*UBE3B, CLTCL1, NCKAP5L, ZNF18*
Timothy W. Yu, et al. Neuron 2013 [7] (*n* = 6)	*AMT, PEX7, SYNE1, VPS13B, PAH, POMGNT1*

Variants in *VPS13B* in this study and in Yu et al [7] are the same ones annotated by different transcripts.

Table S4. ASD-associated DNGs sourced from the Gene4denovo database.

Gene	DNV rate	PTV	Dmis	PTV+Dmis	P-value	FDR
CHD8	9.79E-05	10	4	14	2.21E-06	1.97E-13
SCN2A	7.84E-05	5	7	12	2.21E-06	9.20E-11
SYNGAP1	6.85E-05	8	1	9	2.21E-06	2.36E-09
PTEN	1.51E-05	3	6	9	2.21E-06	4.29E-09
KDM5B	6.66E-05	5	4	9	2.21E-06	2.30E-08
SLC6A1	3.14E-05	3	5	8	2.21E-06	7.90E-08
ADNP	4.12E-05	6	0	6	2.21E-06	6.62E-07
SHANK3	5.90E-05	6	0	6	2.21E-06	1.98E-06
ARID1B	9.42E-05	6	1	7	2.21E-06	3.07E-06
DYRK1A	3.32E-05	5	0	5	2.21E-06	8.17E-06
CHD2	7.59E-05	5	1	6	2.21E-06	1.50E-05
GRIN2B	7.56E-05	4	2	6	2.21E-06	3.17E-05
ANK2	1.57E-04	5	2	7	2.21E-06	8.72E-05
TBR1	3.65E-05	2	3	5	2.21E-06	0.00014461
NAA15	3.06E-05	4	0	4	2.21E-06	0.0001966
MYO1E	5.07E-05	3	2	5	2.21E-06	0.00024505
POGZ	6.02E-05	3	2	5	4.42E-06	0.00032561
PPP2R5D	2.84E-05	3	1	4	4.42E-06	0.00042944
FOXP1	3.24E-05	3	1	4	4.42E-06	0.00055067
DNMT3A	5.04E-05	1	4	5	8.84E-06	0.0006939
KMT5B	3.69E-05	3	1	4	8.84E-06	0.00082699
TRIP12	7.91E-05	3	2	5	8.84E-06	0.00098853
STXBP1	3.12E-05	1	3	4	2.21E-05	0.00142246
WAC	2.60E-05	3	0	3	2.65E-05	0.00192332
WDFY3	1.43E-04	3	3	6	2.65E-05	0.0023957
BRD7	2.69E-05	3	0	3	2.65E-05	0.00284594
PIK3CA	3.99E-05	0	4	4	4.86E-05	0.00380694
ASB14	1.12E-05	1	2	3	5.30E-05	0.00479692
NR2F1	2.67E-05	2	1	3	5.74E-05	0.00582972
DSCAM	1.07E-04	4	0	4	7.07E-05	0.00695351
KCNQ2	4.82E-05	3	0	3	7.51E-05	0.00805176
TCF4	3.04E-05	2	1	3	7.51E-05	0.00911506
DDX3X	3.08E-05	2	1	3	7.95E-05	0.01013902
PBX1	1.88E-05	1	2	3	8.40E-05	0.0112337
GALNT18	3.36E-05	2	1	3	8.40E-05	0.01232138
PRPF8	1.15E-04	2	3	5	0.00010605	0.01346538
ASH1L	1.14E-04	4	0	4	0.00010605	0.01455904
NRXN1	7.67E-05	2	2	4	0.00013257	0.01574812
AGO3	3.78E-05	2	1	3	0.00013699	0.01690486
PRKAR1B	2.40E-05	1	2	3	0.00014141	0.01803582
GRIK1	3.85E-05	2	1	3	0.00014582	0.01912965
GIGYF1	5.89E-05	3	0	3	0.00015908	0.02029605
PTPN11	2.75E-05	1	2	3	0.00017676	0.02154851
LMTK3	4.41E-05	2	1	3	0.00020769	0.02292987
TANC2	8.44E-05	2	2	4	0.00021211	0.0243278
RFX3	3.05E-05	1	2	3	0.00021211	0.02567046

TLK2	3.20E-05	1	2	3	0.0002342	0.02709475
FXYD5	9.30E-06	2	0	2	0.0002342	0.02847481
SET	9.53E-06	2	0	2	0.00024304	0.02982169
GRIA2	3.64E-05	1	2	3	0.00033584	0.03151162
ATP1B1	1.38E-05	2	0	2	0.00034026	0.03315995
NUDT17	1.40E-05	2	0	2	0.00034468	0.0347648
ACHE	3.75E-05	1	2	3	0.00035351	0.03637876
ILF2	1.57E-05	2	0	2	0.00038445	0.0380407
SCN1A	8.00E-05	1	3	4	0.00039328	0.03968943
STK33	1.69E-05	2	0	2	0.00044631	0.0413575
PAPOLG	2.90E-05	0	3	3	0.00046399	0.04300868
FANCE	1.85E-05	2	0	2	0.0004905	0.04473589
MECOM	4.13E-05	1	2	3	0.00049492	0.04640494
LAMB1	8.29E-05	1	3	4	0.00051701	0.04810137
NFE2L3	2.04E-05	2	0	2	0.00057446	0.04986466
KATNAL2	2.14E-05	2	0	2	0.00061865	0.05168167
TFAP2C	2.31E-05	2	0	2	0.00066726	0.05363418
ATP1A1	4.59E-05	1	2	3	0.00066726	0.05553142
ASXL3	8.26E-05	3	0	3	0.00073796	0.05750929
SLC4A9	3.62E-05	0	3	3	0.00076005	0.05948667
SPAST	2.51E-05	2	0	2	0.00076889	0.06143745
CELF2	2.52E-05	2	0	2	0.00077773	0.06333949
EYA1	2.58E-05	2	0	2	0.00080866	0.06525474
CALU	9.31E-06	1	1	2	0.00080866	0.06713448
EPHB1	4.96E-05	1	2	3	0.00086169	0.06905605
OR10Z1	1.08E-05	1	1	2	0.00092355	0.07101154
GGNBP2	2.76E-05	2	0	2	0.00093239	0.07292401
UNC80	1.09E-05	1	1	2	0.00093239	0.07478835
TCF7L2	2.82E-05	2	0	2	0.00094565	0.07666498
ANKRD27	5.12E-05	1	2	3	0.0009589	0.07852827
ERI1	1.19E-05	1	1	2	0.00097216	0.0803642
RPSA	1.21E-05	1	1	2	0.00099867	0.08217569
SLC6A8	3.02E-05	2	0	2	0.0010738	0.08408828
ERGIC2	1.35E-05	1	1	2	0.00109147	0.08596996
RNF146	1.46E-05	1	1	2	0.00116217	0.08793837
CUL3	3.17E-05	2	0	2	0.00116217	0.08987526
CELF4	3.23E-05	2	0	2	0.00118869	0.09183769
QRICH1	3.25E-05	2	0	2	0.00120636	0.09377518
TSPAN4	1.59E-05	1	1	2	0.00122846	0.09571245
TBL1XR1	1.60E-05	1	1	2	0.00123288	0.0976205
USP15	3.31E-05	2	0	2	0.0012373	0.09949008

Genes with FDR < 0.1 were sourced from the Gene4Denovo database [4] we recently developed (http://genemed.tech/gene4denovo/).

Table S5. Functional enrichment of ASD-associated RIGs and DNGs.

Category	*P*-value_85	*P*-value_157	Gene symbols
GO:0016570 histone modification	6.35E-10	1.07E-09	RIGs: *UBE2A*, *CUL4B*, *CPA4*, *RIF1*, *HDAC8*, *BRMS1L*, *DYDC1*<br>DNGs: *MECOM*, *EYA1*, *SET*, *TRIP12*, *USP15*, *KDM5B*, *BRD7*, *KMT5B*, *WAC*, *ASH1L*, *TBL1XR1*
GO:0051603 proteolysis involved in cellular protein catabolic process	1.41E-06	2.76E-04	RIGs: *UBE2A*, *USP9X*, *CUL4B*, *NUPR1*, *CCDC22*, *UBE3B*<br>DNGs: *PTEN*, *CUL3*, *TRIP12*, *USP15*, *TLK2*, *WAC*, *TBL1XR1*, *RNF146*
GO:0070997 neuron death	4.16E-06	2.02E-05	RIGs: *ATG7*, *NUPR1*, *NCOA7*<br>DNGs: *EPHB1*, *GRIN2B*, *PIK3CA*, *SCN2A*, *SET*, *STXBP1*, *SYNGAP1*, *ADNP*
GO:0000904 cell morphogenesis involved in differentiation	2.10E-06	1.96E-06	RIGs: *LLGL1*, *USP9X*<br>DNGs: *DSCAM*, *EPHB1*, *LAMB1*, *PIK3CA*, *PTEN*, *PTPN11*, *SPAST*, *STXBP1*, *CUL3*, *SYNGAP1*, *NRXN1*, *TBR1*, *ADNP*, *TANC2*, *ANKRD27*, *SHANK3*
GO:0007268 chemical synaptic transmission	2.03E-04	4.30E-08	RIGs: *CA2*, *SLC1A1*, *FCHSD2*, *CDHR3*<br>DNGs: *ACHE*, *EPHB1*, *GRIA2*, *GRIK1*, *GRIN2B*, *KCNQ2*, *PRKAR1B*, *PTEN*, *SLC6A1*, *STXBP1*, *SYNGAP1*, *NRXN1*, *ADNP*, *CELF4*, *ARID1B*, *SHANK3*
FMRP targets	4.05E-10	2.96E-11	RIGs: *GCN1L1*, *HIPK3*, *LLGL1*, *SYNE1*, *UBE3B*, *USP9X*<br>DNGs: *ADNP*, *ANK2*, *ASH1L*, *ATP1A1*, *ATP1B1*, *CHD8*, *DSCAM*, *GRIN2B*, *KCNQ2*, *LMTK3*, *NR2F1*, *NRXN1*, *PRPF8*, *PTEN*, *PTPN11*, *SHANK3*, *SLC6A1*, *STXBP1*, *SYNGAP1*, *TANC2*, *TCF4*, *TRIP12*, *WDFY3*
Essential genes	5.66E-09	2.69E-09	RIGs: *ATG7*, *COL19A1*, *FEV*, *DAC8*, *KIF22*, *LLGL1*, *MED12*, *PEX7*, *POMGNT1*, *RIF1*, *SYNE1*, *UBE2A*<br><br>DNGs: *ACHE*, *ADNP*, *ANK2*, *ATP1A1*, *CELF4*, *CHD2*, *CHD8*, *CUL3*, *DNMT3A*, *SCAM*, *DYRK1A*, *ERI1*, *EYA1*, *FOXP1*, *GRIA2*, *GRIN2B*, *ILF2*, *KCNQ2*, *LAMB1*, *MECOM*, *MYO1E*, *NR2F1*, *PBX1*, *PIK3CA*, *PPP2R5D*, *PTEN*, *PTPN11*, *RFX3*, *RPSA*, *SCN1A*, *SCN2A*, *STXBP1*, *SYNGAP1*, *TANC2*, *TBR1*, *TCF4*, *TCF7L2*, *TFAP2C*

P-value_85, the P-value for the 85 genes in the functional network; P-value_157, the P-value for all 157 DNGs and RIGs integrated in this study. Genes contained in the functional network in Figure 2 are underlined.

Table S6. Expression patterns of 70 RIGs and 87 DNGs in ASD.

Gene symbol	Types	Spatio-temporal expression patterns	Prenatal neocortical	Inhibitory and excitatory neuronal expression pattern
ABCC10	RIGs	M0	Ma	Not significant
AFF2	RIGs	M1	Ma	Inhibitory neuronal significant
AMT	RIGs	M2	Mb	Not significant
ANO5	RIGs	M2	Mc	Not significant
APOO	RIGs	M0	Mo	Inhibitory neuronal significant
ATAD3A	RIGs	M0	Mo	Not significant
ATG7	RIGs	M0	Mb	Excitatory neuronal significant
ATP9B	RIGs	M2	Mb	Not significant
BRMS1L	RIGs	M2	Mc	Not significant
BTAF1	RIGs	M1	Mc	Excitatory neuronal significant
CA2	RIGs	M2	Mo	Inhibitory neuronal significant
CCDC22	RIGs	M0	Mb	Excitatory neuronal significant
CDHR3	RIGs	M1	Ma	Excitatory neuronal significant
CGN	RIGs	M2	Mo	Inhibitory neuronal significant
CHTF18	RIGs	M1	Mb	Excitatory neuronal significant
CLTCL1	RIGs	M1	Ma	Not significant
COL19A1	RIGs	M0	Ma	Not significant
CPA4	RIGs	M2	Mo	Excitatory neuronal significant
CUL4B	RIGs	M1	Mc	Inhibitory neuronal significant
CXCL9	RIGs	M0	Mo	Not significant
CYBB	RIGs	M0	Mb	Not significant
DDHD1	RIGs	M2	Mc	Excitatory neuronal significant
DNAI2	RIGs	M0	Mo	Excitatory neuronal significant
DYDC1	RIGs	M0	Ma	Excitatory neuronal significant
ELOF1	RIGs	M0	Mo	Not significant
EML5	RIGs	M1	Mc	Not significant
FCHSD2	RIGs	M1	Ma	Inhibitory neuronal significant
FEV	RIGs	M2	Mo	Excitatory neuronal significant
GCN1L1	RIGs	M1	Mb	Not significant
GRIPAP1	RIGs	M2	Ma	Not significant
GZF1	RIGs	M2	Mc	Inhibitory neuronal significant
HDAC8	RIGs	M0	Mo	Not significant
HIPK3	RIGs	M2	Mo	Not significant
HOXC5	RIGs	M0	Mo	Not significant
IFITM5	RIGs	M0	Ma	Not significant
IL13RA1	RIGs	M0	Mb	Not significant
INTS4	RIGs	M1	Mo	Not significant
ITPRIPL1	RIGs	M1	Mb	Excitatory neuronal significant
KIF22	RIGs	M1	Mb	Inhibitory neuronal significant
LLGL1	RIGs	M0	Mo	Not significant
LSMEM1	RIGs	M0	Mo	Not significant
MED12	RIGs	M1	Mo	Not significant
MEDAG	RIGs	M0	Mo	Excitatory neuronal significant
MOB1B	RIGs	M1	Mc	Not significant
NCKAP5L	RIGs	M1	Ma	Inhibitory neuronal significant

NCOA7	RIGs	M2	Ma	Excitatory neuronal significant
NSUN2	RIGs	M1	Mo	Excitatory neuronal significant
NUPR1	RIGs	M0	Mb	Not significant
PAH	RIGs	M2	Mo	Not significant
PEX7	RIGs	M2	Mc	Not significant
POMGNT1	RIGs	M0	Ma	Not significant
RARB	RIGs	M0	Mo	Not significant
RBMX	RIGs	M1	Mb	Inhibitory neuronal significant
RFT1	RIGs	M0	Mb	Not significant
RFX5	RIGs	M2	Mb	Inhibitory neuronal significant
RIF1	RIGs	M1	Mc	Inhibitory neuronal significant
ROGDI	RIGs	M2	Ma	Excitatory neuronal significant
SCGB1D1	RIGs	M0	Mo	Not significant
SLC1A1	RIGs	M0	Ma	Inhibitory neuronal significant
SLC22A6	RIGs	M0	Mo	Excitatory neuronal significant
SLC35E3	RIGs	M1	Mc	Not significant
SLC38A5	RIGs	M0	Mb	Not significant
SYNE1	RIGs	M2	Ma	Not significant
UBE2A	RIGs	M0	Mc	Not significant
UBE3B	RIGs	M2	Mo	Not significant
USH2A	RIGs	M2	Mo	Excitatory neuronal significant
USP9X	RIGs	M1	Ma	Not significant
VPS13B	RIGs	M1	Mo	Not significant
ZNF16	RIGs	M1	Mo	Not significant
ZNF18	RIGs	M1	Ma	Excitatory neuronal significant
ACHE	DNGs	M2	Ma	Inhibitory neuronal significant
ADNP	DNGs	M1	Mc	Inhibitory neuronal significant
AGO3	DNGs	M1	Mo	Excitatory neuronal significant
ANK2	DNGs	M0	Mo	Excitatory neuronal significant
ANKRD27	DNGs	M1	Ma	Excitatory neuronal significant
ARID1B	DNGs	M1	Mo	Not significant
ASB14	DNGs	M1	Mo	Not significant
ASH1L	DNGs	M2	Mo	Not significant
ASXL3	DNGs	M1	Ma	Excitatory neuronal significant
ATP1A1	DNGs	M2	Ma	Inhibitory neuronal significant
ATP1B1	DNGs	M2	Mo	Inhibitory neuronal significant
BRD7	DNGs	M1	Mb	Not significant
CALU	DNGs	M1	Mb	Not significant
CELF2	DNGs	M1	Mc	Excitatory neuronal significant
CELF4	DNGs	M2	Ma	Excitatory neuronal significant
CHD2	DNGs	M1	Mo	Not significant
CHD8	DNGs	M1	Ma	Not significant
CUL3	DNGs	M1	Ma	Not significant
DDX3X	DNGs	M1	Mo	Not significant
DNMT3A	DNGs	M1	Mo	Excitatory neuronal significant
DSCAM	DNGs	M1	Ma	Excitatory neuronal significant
DYRK1A	DNGs	M1	Mc	Excitatory neuronal significant
EPHB1	DNGs	M1	Ma	Not significant
ERGIC2	DNGs	M0	Mc	Inhibitory neuronal significant

ERI1	DNGs	M1	Mc	Excitatory neuronal significant
EYA1	DNGs	M0	Mo	Not significant
FANCE	DNGs	M1	Ma	Inhibitory neuronal significant
FOXP1	DNGs	M0	Ma	Excitatory neuronal significant
FXYD5	DNGs	M0	Mb	Excitatory neuronal significant
GALNT18	DNGs	M2	Ma	Not significant
GGNBP2	DNGs	M1	Ma	Excitatory neuronal significant
GIGYF1	DNGs	M0	Mo	Not significant
GRIA2	DNGs	M2	Ma	Excitatory neuronal significant
GRIK1	DNGs	M2	Mo	Inhibitory neuronal significant
GRIN2B	DNGs	M0	Ma	Inhibitory neuronal significant
ILF2	DNGs	M1	Mo	Not significant
KATNAL2	DNGs	M2	Mo	Not significant
KCNQ2	DNGs	M1	Mb	Excitatory neuronal significant
KDM5B	DNGs	M1	Mo	Excitatory neuronal significant
KMT5B	DNGs	M1	Ma	Not significant
LAMB1	DNGs	M0	Ma	Excitatory neuronal significant
LMTK3	DNGs	M0	Ma	Inhibitory neuronal significant
MECOM	DNGs	M0	Mo	Not significant
MYO1E	DNGs	M0	Mb	Excitatory neuronal significant
NAA15	DNGs	M1	Mc	Not significant
NFE2L3	DNGs	M1	Ma	Not significant
NR2F1	DNGs	M1	Mo	Not significant
NRXN1	DNGs	M0	Ma	Not significant
NUDT17	DNGs	M0	Mo	Not significant
OR10Z1	DNGs	M0	Mo	Not significant
PAPOLG	DNGs	M1	Mc	Not significant
PBX1	DNGs	M1	Ma	Not significant
PIK3CA	DNGs	M1	Mc	Not significant
POGZ	DNGs	M1	Ma	Excitatory neuronal significant
PPP2R5D	DNGs	M0	Ma	Not significant
PRKAR1B	DNGs	M2	Mo	Excitatory neuronal significant
PRPF8	DNGs	M1	Mo	Inhibitory neuronal significant
PTEN	DNGs	M1	Mc	Excitatory neuronal significant
PTPN11	DNGs	M2	Mo	Excitatory neuronal significant
QRICH1	DNGs	M1	Ma	Not significant
RFX3	DNGs	M1	Mc	Not significant
RNF146	DNGs	M1	Mc	Excitatory neuronal significant
RPSA	DNGs	M1	Ma	Not significant
SCN1A	DNGs	M2	Ma	Inhibitory neuronal significant
SCN2A	DNGs	M2	Ma	Excitatory neuronal significant
SET	DNGs	M1	Mo	Not significant
SHANK3	DNGs	M2	Ma	Not significant
SLC4A9	DNGs	M2	Ma	Not significant
SLC6A1	DNGs	M2	Mb	Inhibitory neuronal significant
SLC6A8	DNGs	M2	Mb	Not significant
SPAST	DNGs	M1	Mc	Not significant
STK33	DNGs	M0	Mb	Excitatory neuronal significant
STXBP1	DNGs	M2	Ma	Excitatory neuronal significant

SYNGAP1	DNGs	M0	Mo	Excitatory neuronal significant
TANC2	DNGs	M1	Ma	Excitatory neuronal significant
TBL1XR1	DNGs	M1	Mc	Excitatory neuronal significant
TBR1	DNGs	M1	Ma	Excitatory neuronal significant
TCF4	DNGs	M1	Mo	Inhibitory neuronal significant
TCF7L2	DNGs	M0	Mb	Excitatory neuronal significant
TFAP2C	DNGs	M1	Mb	Not significant
TLK2	DNGs	M1	Mo	Not significant
TRIP12	DNGs	M1	Mc	Excitatory neuronal significant
TSPAN4	DNGs	M0	Ma	Inhibitory neuronal significant
UNC80	DNGs	M2	Ma	Excitatory neuronal significant
USP15	DNGs	M1	Ma	Excitatory neuronal significant
WAC	DNGs	M2	Mc	Excitatory neuronal significant
WDFY3	DNGs	M0	Mc	Not significant

Genes contained in M0 and Mo were not clustered into any co-expression modules by weighted correlation network analysis.

Table S7. Comparison of expression patterns between DNGs and RIGs in ASD, ID and CHD.

**Disorder**	**Gene (*n*)**	Spatio-temporal				Prenatal neocortical			Exc and Inh neurons		
		M1	M2	M0	Ma	Mb	Mc	Mo	Exc	Inh	Not sig
	DNGs (87)	47	20	20	35	10	16	26	35	14	38
	RIGs (70)	23	20	27	17	15	12	26	18	13	39
ASD-Wang	P-value	**2.21E-02**	0.46	-	**4.13E-02**	0.12	1	-	0.063	0.83	-
	OR	2.19	0.75	-	2.09	0.48	1.09	-	1.94	0.84	-
	95% CI	1.08-4.49	0.34-1.64	-	1.00-4.50	0.18-1.24	0.44-2.74	-	0.93-4.12	0.34-2.12	-
	DNGs (102)	66	18	18	61	9	13	28	39	22	41
	RIGs (70)	25	19	26	23	14	12	35	18	13	39
ASD-Satterstrom	P-value	**2.02E-04**	0.19	-	**6.25E-04**	**0.041**	0.51	-	0.10	0.7	-
	OR	3.28	0.58	-	3.02	0.39	0.71	-	1.78	1.2	-
	95% CI	1.67-6.56	0.26-1.28	-	1.54-6.06	0.14-1.39	0.28-1.83	-	0.88-3.72	0.53-2.83	-
	DNGs (82)	45	18	19	31	13	13	25	25	15	42
	RIGs (120)	51	26	43	41	15	13	51	21	29	70
ID	P-value	0.088	1	-	0.65	0.54	0.39	-	**0.040**	0.39	-
	OR	1.64	1.02	-	1.17	1.32	1.55	-	2.06	0.7	-
	95% CI	0.90-3.01	0.48-2.11	-	0.62-2.19	0.54-3.18	0.62-3.86	-	1.01-4.26	0.32-1.48	-
	DNGs (78)	42	14	22	21	18	12	27	28	12	38
	RGs (88)	43	17	28	23	24	15	26	32	12	44
CHD	P-value	0.53	0.84	-	1	0.59	0.84	-	1	0.83	-
	OR	1.22	0.91	-	1.04	0.8	0.89	-	0.98	1.15	-
	95% CI	0.63-2.35	0.38-2.15	-	0.49-2.20	0.37-1.71	0.35-2.19	-	0.49-1.94	0.44-3.01	-

ASD, autism spectrum disorder; ID, intellectual disability; CHD, congenital heart defects; Exc, excitatory neurons; Inh, inhibitory neurons; OR, odds ratio; CI, confidence interval; RIGs, recessive inherited genes; DNGs, genes with *de novo* variants. In the panels of ASD-Wang and ASD-Satterstrom, the RIGs were sourced from Table S3 and the DNGs were sourced from the Gene4Denovo database [4] and Satterstrom et al [8], respectively. In the ID panel, the RIGs were sourced from the OMIM database, and the DNGs were sourced from the Gene4Denovo database [4]. In the CHD panel, the RIGs were sourced from Jin et al [9] and the DNGs were sourced from Gene4Denovo database [4]. Genes in M0 and Mo were not clustered into any co-expression modules by weighted correlation network analysis. We used Fisher's exact test to perform enrichment analysis. P-values <0.05 are highlighted in bold.

References:

1. Deciphering Developmental Disorders S. Prevalence and architecture of de novo mutations in developmental disorders. Nature. 2017;542:433-438.
2. Lelieveld SH, Reijnders MR, Pfundt R, Yntema HG, Kamsteeg EJ, de Vries P, et al. Meta-analysis of 2,104 trios provides support for 10 new genes for intellectual disability. Nat Neurosci. 2016;19:1194-1196.
3. Iossifov I, O'Roak BJ, Sanders SJ, Ronemus M, Krumm N, Levy D, et al. The contribution of de novo coding mutations to autism spectrum disorder. Nature. 2014;515:216-221.
4. Zhao G, Li K, Li B, Wang Z, Fang Z, Wang X, et al. Gene4Denovo: an integrated database and analytic platform for de novo mutations in humans. Nucleic Acids Res. 2020;48:D913-D926.
5. Doan RN, Lim ET, De Rubeis S, Betancur C, Cutler DJ, Chiocchetti AG, et al. Recessive gene disruptions in autism spectrum disorder. Nat Genet. 2019;51:1092-1098.
6. Chahrour MH, Yu TW, Lim ET, Ataman B, Coulter ME, Hill RS, et al. Whole-exome sequencing and homozygosity analysis implicate depolarization-regulated neuronal genes in autism. PLoS Genet. 2012;8:e1002635.
7. Yu TW, Chahrour MH, Coulter ME, Jiralerspong S, Okamura-Ikeda K, Ataman B, et al. Using whole-exome sequencing to identify inherited causes of autism. Neuron. 2013;77:259-273.
8. Satterstrom FK, Kosmicki JA, Wang J, Breen MS, De Rubeis S, An JY, et al. Large-Scale Exome Sequencing Study Implicates Both Developmental and Functional Changes in the Neurobiology of Autism. Cell. 2020;180:568-584 e523.
9. Jin SC, Homsy J, Zaidi S, Lu Q, Morton S, DePalma SR, et al. Contribution of rare inherited and de novo variants in 2,871 congenital heart disease probands. Nat Genet. 2017;49:1593-1601.
